# Transfer of IGF2BP3 Through Ara-C-Induced Apoptotic Bodies Promotes Survival of Recipient Cells

**DOI:** 10.3389/fonc.2022.801226

**Published:** 2022-05-09

**Authors:** Junjie Gou, Hongjiao Li, Jingjing Bi, Xingchen Pang, Xiang Li, Yi Wang

**Affiliations:** ^1^Key Laboratory of Resource Biology and Biotechnology in Western China, Ministry of Education, Provincial Key Laboratory of Biotechnology, College of Life Sciences, Northwest University, Xi’an, China; ^2^Institute of Hematology, School of Medicine, Northwest University, Xi’an, China; ^3^Department of Hematology, Provincial People’s Hospital, Xi’an, China

**Keywords:** apoptotic bodies, IGF2 mRNA-binding protein 3 (IGF2BP3), cytosine arabinoside (Ara-C), myelodysplastic syndromes (MDS), acute myeloid leukemia (AML)

## Abstract

Cytosine arabinoside (Ara-C) has been the standard therapeutic agent for myelodysplastic syndromes (MDS) and adult acute myeloid leukemia (AML) patients for decades. Considerable progress has been made in development of new treatments for MDS/AML patients, but drug resistance remains a major clinical problem. Apoptotic bodies (ABs), produced by late apoptotic cells, can enclose bioactive components that affect cell-cell interactions and disease progression. We isolated and identified drug-induced ABs from Ara-C-tolerance cells. Treatment of sensitive cells with Ara-C-induced ABs resulted in Ara-C-resistant phenotype. We further investigated components and functions of Ara-C-induced ABs. Proteomics analysis in combination with mass spectrometry revealed that Ara-C-induced ABs carried numerous RNA-binding proteins, notably including insulin-like growth factor 2 mRNA-binding protein 3 (IGF2BP3). Delivery of AB-encapsulated IGF2BP3 promoted survival of recipient cells by activating PI3K-AKT and p42-44 MAPK pathways. High IGF2BP3 level in ABs from MDS/AML patient plasma was correlated with poor overall survival. Our findings demonstrate that AB-derived IGF2BP3 plays an essential role in acquired Ara-C resistance in MDS/AML patients, and is a potential therapeutic target for suppression of Ara-C resistance.

## Introduction

Myelodysplastic syndromes (MDS) comprise a heterogeneous group of myeloid neoplasms characterized by peripheral blood cytopenia, hematopoietic cell dysplasia, and variable disease course. ~30% of MDS patients are at risk of transformation to secondary acute myeloid leukemia (AML) (1). Despite major advances in recent decades in our understanding of the molecular bases of hematopoietic malignancy and development of mutation targeted therapies, survival rates for AML have not improved significantly, and standard therapy still involves highly toxic chemotherapy and allogeneic hematopoietic cell transplantation (2, 3). ~60% of AML patients respond favorably to these treatments and undergo complete remission; however, a smaller proportion eventually relapse, develop refractory disease or chemoresistance, and survive for less than five years (4). The main obstacle to successful AML treatment is drug resistance that occurs initially in leukemic cells (primary resistance) or that develops during or after treatment (acquired resistance) (5). Genetic, molecular, and cellular mechanisms underlying acquired drug resistance have been described.

Increasing evidence during the past decade has demonstrated the involvement of extracellular vesicles (EVs) in resistance to cancer therapies (6–8). EVs are a type of nano-sized particles that originate from the endosomal pathway and are secreted into surrounding extracellular space by exocytosis. They are classified on the basis of diameter into exosomes, microvesicles, and apoptotic bodies (ABs). Diameters of ABs range from 1-5 μm (9–11). Two physiological processes in which ABs play important roles are apoptotic cell clearance and intercellular communication (12). They contain a variety of bioactive molecules, including DNAs, RNAs, and proteins, which are involved in communication with surrounding cells (13, 14). In a mouse bone defect model, for example, ABs produced by osteoclasts induced endothelial progenitor cell differentiation and promoted CD31 cell production by carrying PDGF-BB protein (15). In a myocardial infarction (MI) model, transplanted mesenchymal stem cells released ABs that enhanced angiogenesis and cardiac function recovery by regulating autophagy of receptor endothelial cells by macrophages (16). It remains unclear whether ABs produced by tumor cells can affect chemotherapeutic treatments of MDS/AML patients.

We examined functional differences between ABs produced by serum starvation vs. cytosine arabinoside (Ara-C) treatment of malignant hematopoietic cells, and treated recipient cells with the two types of ABs. Proliferation and chemotherapy tolerance of recipient cells were notably enhanced by treatment with Ara-C-induced ABs.

## Materials and Methods

### Cell Lines and Culture

Human hematopoietic cell lines KG1a, SKM1 and OCI-AML3 were grown and propagated as described previously (17). Human leukemia-derived cell line ML-1 was kindly donated by Dr. H.J. Deeg (Fred Hutchinson Cancer Research Center; Seattle, WA, USA). All cells were cultured in RPMI 1640 medium (HyClone; Provo, UT, USA) supplemented with 10% fetal bovine serum (Biological Industries; Kibbutz Beit-Haemek, Israel), and 1% penicillin/streptomycin (Gibco; Carlsbad, CA, USA) at 37°C in 5% CO2 atmosphere.

### CCK8 Assay

Cell viability was measured using CCK-8 kit (TopScience; Shanghai, China) as per manufacturer’s protocol. In brief, 10,000 cells were seeded onto 96-well plate, incubated overnight under permissive conditions, induced for 48 h by various concentrations of Ara-C, and treated with 20 µL CCK-8 solution for 1 h at 37°C. Absorbance at wavelength 450 nm was measured by microplate reader (Bio-Rad Laboratories, Hercules, CA, USA). Half maximal inhibitory concentration (IC_50_) was calculated from survival curves by Bliss method (18).

### Extraction of ABs by Differential Ultracentrifugation

Cells were starved in FBS-free medium or 5 μM Ara-C (Sigma-Aldrich; St. Louis, MO, USA) for 48 h, and centrifuged at 800 x *g* for 10 min. Supernatant was filtered with 5-μm filter membrane to remove large cell debris, and centrifuged at 14,000 x *g* for 30 min. Precipitates were collected, resuspended in sterile PBS, and stored at -80°C. ABs from serum starvation and Ara-C induction were respectively termed AB0 and AB+.

### Isolation of Plasma ABs

For each patient, 5 mL bone marrow aspirate was collected in EDTA anticoagulation tube, and centrifuged at 2000 x *g* for 20 min at 4°C, using bone marrow sample separation kit (TBD; Tianjin, China). 1 mL upper clear plasma was collected and centrifuged at 1500 x *g* for 20 min at 4°C to remove platelets. Remaining supernatant was centrifuged at 14,000 x *g* for 30 min to obtain ABs.

### Transmission Electron Microscopy (TEM)

Purified ABs were perfused and fixed with EM fixative (4% paraformaldehyde + 4% glutaraldehyde), placed on a carbon-coated copper mesh, and immersed in 2% phosphotungstic acid solution. ABs were observed by TEM (model H-7650; Hitachi, Tokyo) at 80 kV.

### Nanoparticle Tracking Analysis

ABs were loaded into NanoSight LM10 instrument (Malvern Instruments; Malvern, UK), and analyzed using NanoSight nanoparticle tracking software program.

### Apoptosis Analysis

SKM1 and ML-1 cells were incubated with AB0 or AB+ for 8 h, treated with 1 or 2 μM Ara-C for 48 h, incubated with 5 μL annexin V-APC (BioLegend; San Diego, CA, USA) and 5 μg/mL 7-AAD (BioLegend) for 15 min, and subjected to flow cytometry (ACEA Biosciences; San Diego).

### Cell Proliferation and Cell Cycle Analyses

For proliferation analysis, cells were treated with 10 μmol/L EdU (GeneCopoeia; CA, USA) for 4 h, harvested, and fixed with 4% (m/V) paraformaldehyde. EdU fluorescence signal was detected by flow cytometry. For cell cycle analysis, EdU-labeled cells were treated with PI/RNase kit (Beyotime Institute of Biotechnology; Haimen, China) for 30 min and subjected to flow cytometry.

### Total Protein Extraction

Cells were collected, washed with PBS three times, lysed with RIPA buffer (50 mM Tris, pH 7.2, 1% Triton X-100, 0.5% sodium deoxycholate, 0.1% SDS, 150 mM NaCl, 10 mM MgCl_2_, 2.5% glycerol) supplemented with protease inhibitor, and centrifuged. Supernatant was collected and subjected to BCA assay (Beyotime).

### Western Blotting Analysis

Protein samples were loaded onto SDS-PAGE gel, separated by electrophoresis, and transferred onto PVDF membrane. Membrane was sealed with 3% (m/v) bovine serum albumin (BSA; Sigma-Aldrich) in TBST (20 mM Tris-HCl, 150 mM NaCl, 0.05% Tween 20, pH 8.0) for 1 h, incubated with primary antibodies overnight at 4°C, rinsed with TBST, and incubated with HRP-labeled secondary antibody (1: 5,000; Beyotime) for 1 h at room temperature. Protein bands were revealed by enhanced chemiluminescence (ECL; Vazyme Biotech; Nanjing, China). Antibodies used: histone 2B (sc-515808, 1: 500), complement component 3 (sc-282289, 1: 1000), insulin-like growth factor 2 mRNA-binding protein 3 (IGF2BP3) (sc-365640, 1: 500), and c-Myc (sc-40, 1: 500) were from Santa Cruz Biotechnology (Santa Cruz, CA, USA). Caspase 3 (#9662, 1: 1000), histone 3 (#4499, 1: 1000), PI3K (#4255, 1: 1000), p-PI3K (#17366, 1: 1000), AKT (#4685, 1: 1000), p-AKT (#4060, 1: 1000), ERK (#4695, 1: 3000), p-ERK (#4370, 1: 3000), and BCL2 (#15071, 1: 1000) were from Cell Signaling Technology (Beverly, MA, USA). GAPDH (G9545, 1: 1000) was from Sigma-Aldrich.

### Confocal Microscopic Analysis

Confocal microscopy plates were pretreated as per specifications for poly-D-lysine hydrobromide (Solarbio Science & Technology; Beijing, China). SKM1 and ML-1 cells were transferred to the pretreated plates. ABs labeled by PKH67 Green Fluorescent Cell Linker Kit (Umibio, Shanghai, China) were added to the plates for 1 h. Cells were washed twice with PBS to remove ABs not fully ingested, fixed with 4% paraformaldehyde for 20 min, incubated with 0.2% Triton X-100 in PBS for 30 min, and incubated with blocking buffer (3% BSA in PBS) for 2 h. Blocking solution was removed, and nuclei and cytoskeleton were stained respectively with DAPI and phalloidin (Abcam; Cambridge, MA, USA) for 15 min. Fluorescence images were obtained by confocal microscopy (model TCS SP8; Leica; Mannheim, Germany).

### Patient Samples

Plasma samples from untreated MDS/AML patients and MDS/AML-DR patients were obtained from Shaanxi Provincial People’s Hospital. Written informed consent was obtained from all patients in accordance with the Declaration of Helsinki. Experiments involving human tissues were approved by the Research Ethics Committee of Northwest University.

### Enzyme-Linked Immunosorbent Assay (ELISA)

Plasma ABs lysed with RIPA buffer were diluted with PBS 1:50, incubated in 96-well ELISA plate for 2 h at 37°C, blocked with 3% BSA for 1 h at room temperature, washed with PBS, added with IGF2BP3 antibody (1:500), and incubated overnight at 4°C. Primary antibody was removed, secondary antibody was added, and ABs were incubated for 1 h at room temperature and washed with PBS. 200 μL tetramethylbenzidine (TMB; Beyotime) was added to each well, reaction was terminated by adding 50 μL of 2 M sulfuric acid, and absorbance at 450 nm was measured as described above.

### Phagocytosis of ABs

Purified ABs were labeled with annexin V-APC as per manufacturer’s protocol, suspended in binding buffer, incubated with annexin V for 30 min, and filtered twice with 10-kD filter membrane. Annexin V-labeled ABs were collected by centrifugation, added to recipient cells for 1 h, washed with PBS five times, and centrifuged at 500 x *g* for 5 min. Cells were resuspended with 200 μL PBS and subjected to flow cytometry.

### Proteomic Analysis

Proteins (1 mg) from ABs were concentrated and desalted using size-exclusion spin ultrafiltration unit (10 KD; Merck Millipore; Darmstadt, Germany), denatured with 8 M urea, 10 mM dithiothreitol (DTT), and 20 mM iodoacetamide (IAM; Sigma-Aldrich), and digested with lysyl endopeptidase (Wako Pure Chemical; Osaka, Japan) 1:100 (w/w) for 4 h at 37°C, and then with trypsin (Promega; Madison, WI, USA) 1:100 (w/w) overnight at 37°C. Peptides were collected by centrifugation and purified using Oasis HLB cartridges (Waters; Milford, MA, USA). Two-dimensional LC-MS and data analysis were performed using LTQ Orbitrap MS (Thermo Fisher), Byonic software program (Protein Metrics; San Carlos, CA, USA), and MaxQuant software program as described previously (19).

### Mouse Model

BALB/c nude mice were kept in a pathogen-free barrier animal facility. Animal experiments were performed in accordance with guidelines of the Animal Care and Use Committee of Northwest University. Six-to-8-wk-old mice were injected s.c. with 2×10^7^ SKM1 cells, and following tumor formation three experimental groups were i.v. injected respectively with PBS, 50 μg AB0, and 50 μg AB+. Mice were then i.v. injected every other day with 2 mg/kg Ara-C. At the conclusion of experiments, mice were euthanized, and tumor volume and body weight were measured.

### Immunohistochemical Analysis

Paraffin-embedded tumor tissue samples were analyzed by immunohistochemistry. In brief, samples were stained with primary antibody overnight at 4°C, incubated with HRP-conjugated secondary antibody for 1 h at 37°C, sprayed with 3,3’-diaminobenzidine (DAB) staining solution (Abcam) for 10 min at room temperature, and examined by microscopy (model E80i; Nikon; Tokyo, Japan). The quantification is calculated by Image-Pro Plus 6.0.

### Cell Counting

After AB0 and AB+ pre-treated cells for 8 h, 1×10^4^ cells were seeded in six-well plates. Cells were treated with different concentration of Ara-C (0, 1, 2, 4 μM) at 0, 6, 12, 24, 36 and 48 h, respectively. Cell counter (IC 1000; Countstar; Shanghai, China) was to detect the number of viable cells in each group.

### Data Analysis

All experiments were performed independently at least three times. Data were presented as mean ± SD and analyzed using software program GraphPad Prism V. 7.0. Datasets for two groups were compared by two-tailed Student’s t-test. Differences with p< 0.05 were considered statistically significant. Notations in figures: *, p< 0.05; **, p< 0.01; ***, p< 0.001.

## Results

### Generation and Characterization of ABs

**Figure 1:**
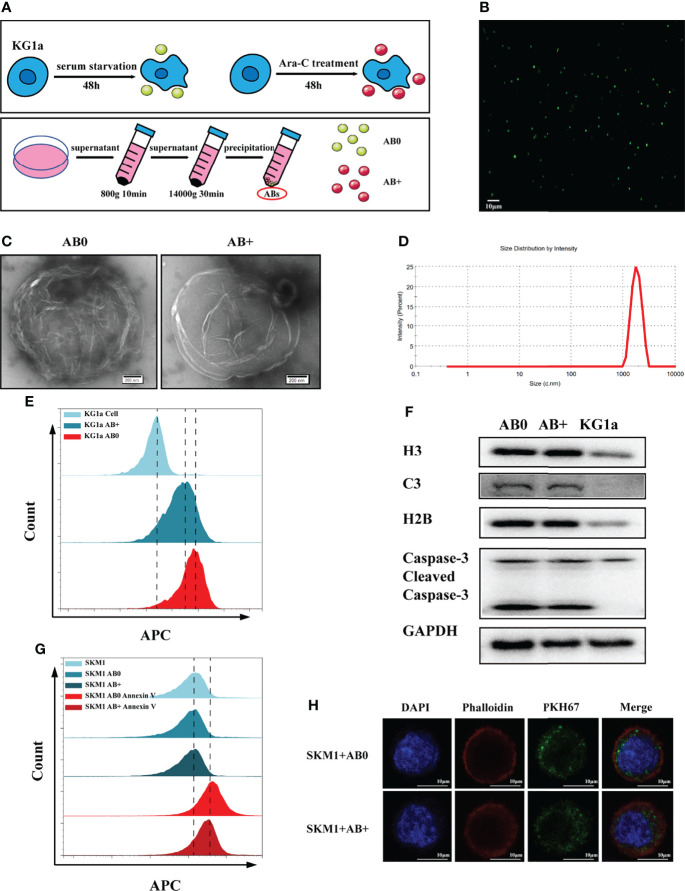
Isolation and characterization of ABs. **(A)** Isolation of ABs from KG1a (schematic). **(B)** Confocal microscopic image of annexin V-stained ABs. Scale bar: 10 µm. **(C)** Vesicular structure of AB0 and AB+ revealed by TEM. Scale bar: 200 nm. **(D)** Size distribution of ABs measured by nanoparticle tracking analysis (NTA). **(E)** AB0, AB+ and live cells stained with annexin V and detected by flow cytometry. **(F)** Histone H3, histone H2B, complement component 3 (C3), caspase 3, cleaved caspase 3, and GAPDH detected by western blotting. **(G)** Annexin V-labeled AB0 and AB+ taken up by SKM1 cells, detected by flow cytometry. **(H)** Confocal microscopic image showing internalization of ABs (dyed with PKH67). Scale bar: 10 µm.

Ara-C tolerance of malignant hematopoietic cell lines (OCI-AML3, KG1a, SKM1 and ML-1) was evaluated by CCK8 assay. Resistance of KG1a was greater than that of SKM1 or ML-1 (**Figure S1A**). KG1a were therefore used as donor cells, and SKM1 and ML-1 as recipient cells, in subsequent experiments. Our hypothesis was that Ara-C resistance can be transferred *via* ABs. ABs were purified from KG1a under serum starvation or under Ara-C treatment without serum for 48 h, and respectively, termed as AB0 and AB+ (**Figure 1A**). Annexin-V-stained ABs were clearly observed by confocal microscopy (**Figure 1B**), and revealed by TEM to have circular vesicle structure (**Figure 1C**), consistently with previous reports (20, 21). The size range of ABs was usually 1-4 μm (**Figure 1D**). Platelets from human peripheral blood, which also have size range 1-4 μm, were used as control. 80-90% of ABs purified by differential centrifugation were in a size range similar to that of platelets (**Figure S1B**). >80% of ABs were recognized by annexin V (**Figure 1E**). In comparison with KG1a cells, isolated ABs had higher expression of caspase 3, histone H3, histone H2B, and complement component 3 (**Figure 1F**). ABs were endocytosed into recipient cells and detectable by flow cytometry (**Figure 1G**) and confocal microscopy (**Figures 1H** and **S1C**). Interestingly, AB+ is more easily engulfed by recipient cells (**Figure S1D**). In conclusion, ABs could be purified from KG1a cells and endocytosed by recipient cells.

### Effect of AB+ on Recipient Cell Survival

**Figure 2:**
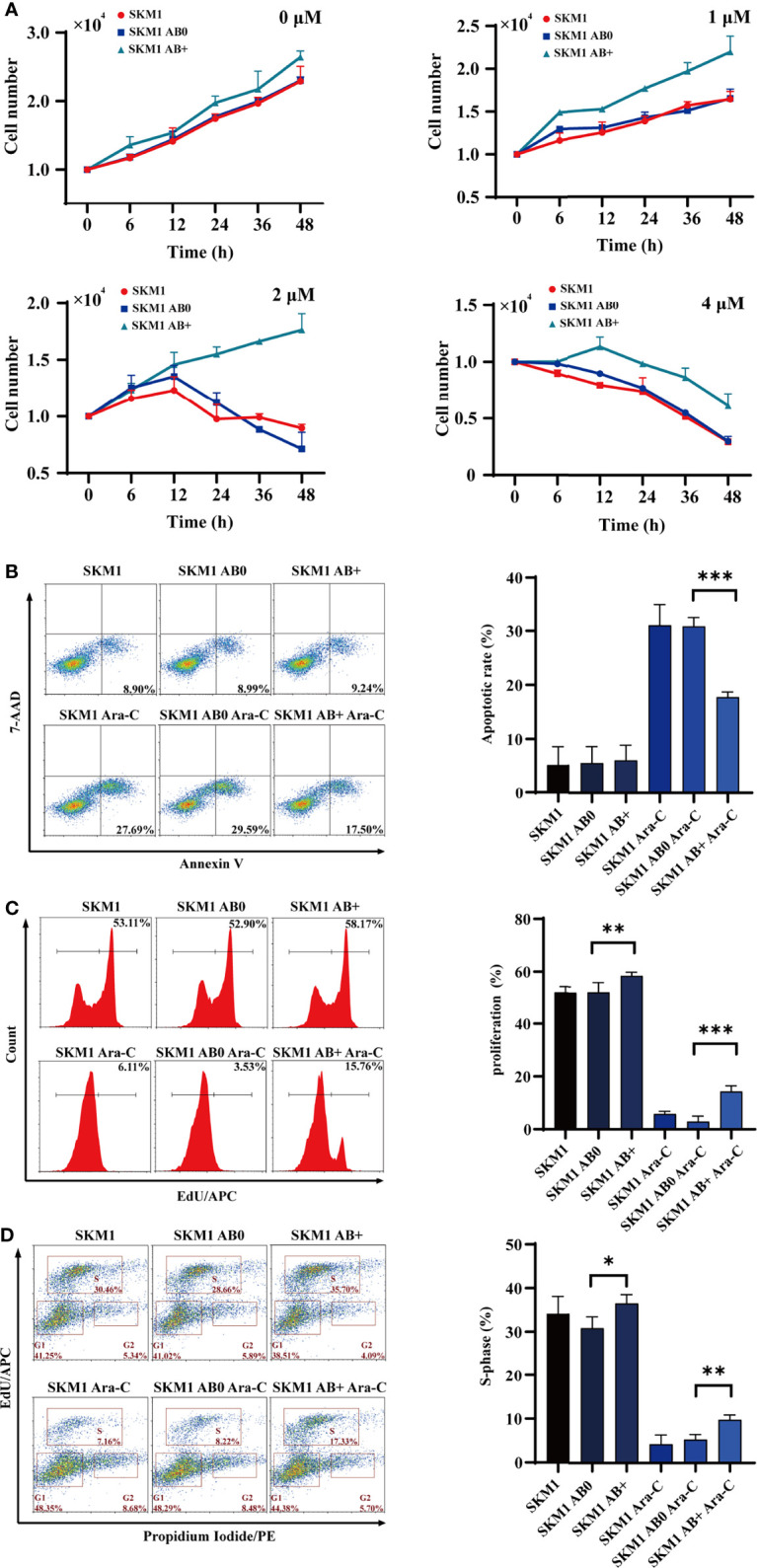
Survival of recipient cells treated with ABs. **(A)** 1×10^4^ SKM1 cells were pre-incubated with AB+ or AB0 for 8 h, and treated with 1, 2, or 4 μM Ara-C for 0-48 h. Cells were counted using cell counter. **(B–D)** SKM1 cells were pre-incubated with AB0 or AB+ for 8 h, then treated with 2 μM Ara-C for 48 h. Controls were untreated cells, and cells treated with AB0 or AB+ but not Ara-C. Apoptosis **(B)**, proliferation **(C)** and cell cycle **(D)** were evaluated by flow cytometry. *p < 0.05; **p < 0.01; ***p < 0.001.

SKM1 and ML-1 cells were treated with 50 μg/mL AB0 or AB+, and then treated with Ara-C at 0, 6, 12, 24, 36, 48 h, respectively. Survival was much higher for AB+-treated vs. AB0-treated cells under various Ara-C doses, suggesting that AB+ treatment enhanced Ara-C resistance and cell growth (**Figure 2A**). AB+-treated cells likewise showed significantly lower apoptosis (**Figures 2B**, **S2A-C**), higher proliferation (**Figures 2C**, **S2D–E**), and promotion of cell cycle G1/S phase (**Figures 2D**, **S2F, G**) relative to AB0-treated cells. Further, we used another cell line OCI-AML3 as donor cells to validate the above results, the similar result was found in recipient SKM1, showing the decreased apoptosis and enhanced cell proliferation (**Figures S3A, B**). To test if the ABs are specific to Ara-C, we incubated cells with doxorubicin (DOX) to collect the ABs. The recipient cells SKM1 after treated with DOX-induced ABs showed lower apoptosis and higher proliferation (**Figures S4A, B**). These data indicated chemotherapeutic drug induced ABs can have drug resistant effect on recipient cells.

### AB+-Encapsulated IGF2BP3 Promotes Survival of Recipient Cells

**Figure 3:**
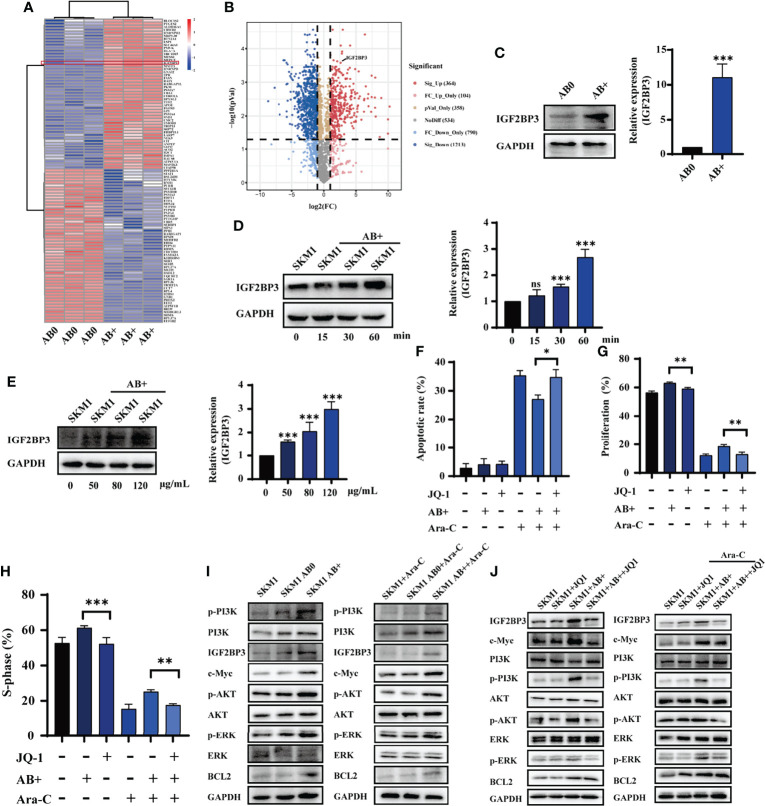
IGF2BP3 from AB+ enhances survival of recipient cells. **(A)** Proteins differentially expressed by AB0 vs. AB+ were identified by mass spectrometry. In this heatmap, the red box indicates differential expression of IGF2BP3. **(B)** Volcano plot showing differential expression (up- and downregulation) of AB0 and AB+ proteins. **(C)** Western blotting analysis of IGF2BP3 expression in AB0 and AB+. Right panel: quantification of results. **(D)** Western blotting analysis of IGF2BP3 expression in SKM1 cells treated with AB+ for various durations. **(E)** Western blotting analysis of IGF2BP3 expression in SKM1 treated for 30 min with various concentrations of AB+. **(F–H)** AB+-treated SKM1 were treated with Ara-C and/or JQ-1 for 48 h, and apoptosis **(F)**, proliferation **(G)**, and cell cycle **(H)** were evaluated. **(I)** Expression of PI3K/AKT and MAPK signaling in untreated, AB0-treated, and AB+-treated SKM1 before and after Ara-C incubation. **(J)** Untreated and AB+-treated SKM1 were incubated with Ara-C and/or JQ-1 for 48 h, and expression of proteins involved in PI3K/AKT and MAPK signaling pathways was evaluated. ns, p > 0.05; *p < 0.05; **p < 0.01; ***p < 0.001.

Proteomic analysis was performed to identify proteins differentially expressed in AB0 vs. AB+. A heatmap and volcano plot, using gating with statistical significance at false discovery rate (FDR) adjusted to p< 0.05 and fold change >2.0, revealed 1577 differentially expressed proteins (**Figure 3A**). Of these, 364 were significant upregulated and 1213 were downregulated in AB+ relative to AB0 (**Figure 3B**). KEGG analysis indicated that the differentially expressed proteins are involved mainly in cell apoptosis and cell cycle (**Figure S5A**). On the other hand, analysis using Metascape database (22) suggested that biological functions of the differentially expressed proteins related mainly to RNA metabolism (**Figure S5B**). Then we counted the differential proteins associated with RNA metabolism (**Figure S5C**). Of particular interest, we observed that expression of insulin growth factor 2 mRNA binding protein 3 (IGF2BP3), a potential oncogene that can induce tumor development (23), was notably higher in AB+ (**Figure S5D**). Western blotting analysis confirmed that IGF2BP3 expression was higher in KG1a than in SKM1 (**Figure S5E**), and higher in AB+ than in AB0 (**Figure 3C**). In AB+-treated SKM1, IGF2BP3 level increased in time- and dose-dependent manners (**Figures 3D, E**) and is mainly distributed in nucleus (**Figure S5F**). When AB+-treated SKM1 were further treated with JQ-1, a bromine terminal inhibitor of IGF2BP3 (24), apoptosis rate was increased, whereas proliferation and S-phase of cell cycle were decreased (**Figures 3F–H**, **S5G**). Specific downstream proteins of IGF2BP3 (c-Myc, p-PI3K, p-ERK anti-apoptotic protein BCL2) were upregulated in AB+-treated SKM1 (**Figure 3I**). Expression of these proteins was lower in JQ-1-treated group than in AB+-treated group (**Figure 3J**). These findings, taken together, indicate that IGF2BP3 delivered by AB+ played a tumor-promoting role in recipient cells.

### AB+-Mediated IGF2BP3 Delivery in a Mouse Model

**Figure 4:**
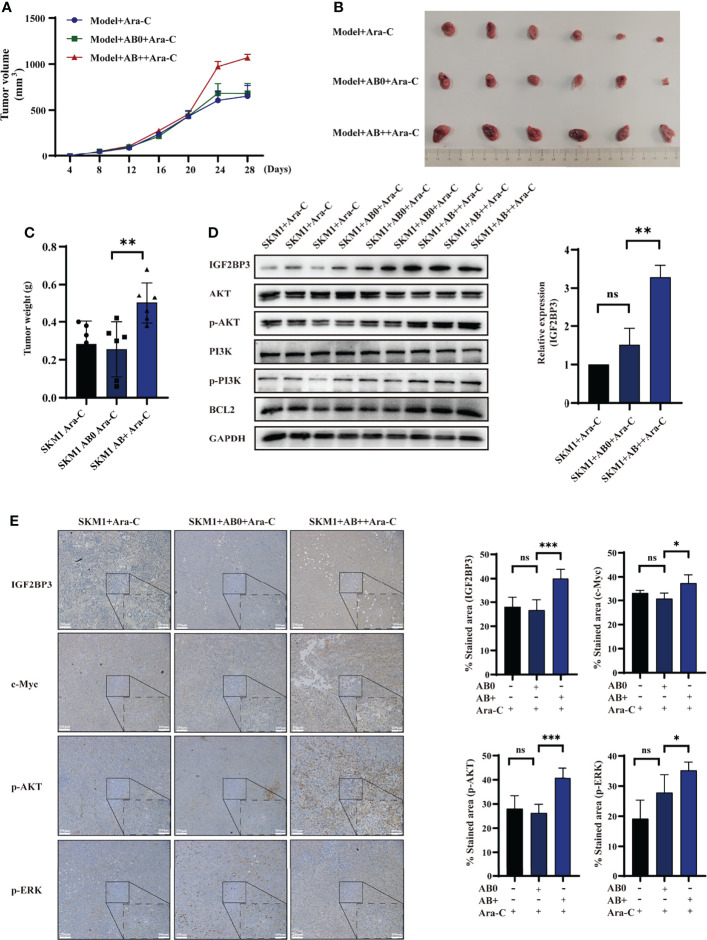
AB+-mediated IGF2BP3 delivery *in vivo*. Mice (n=6 for each group) were pretreated with saline, AB0, or AB+, then injected with 2×10^7^ SKM1 cells. After resulting tumors reached average volume 200 mm^3^, mice were i.v. injected with 2 mg/kg Ara-C every other day. **(A)** Tumor volume were measured at 4-day intervals. **(B, C)** After day 28, tumors were excised, photographed **(B)** and weighed **(C)**. **(D)** Proteins were extracted from tissues, and expression of IGF2BP3, PI3K, p-PI3K, AKT, p-AKT, and BCL2 was analyzed by western blotting, with GAPDH as loading control. **(E)** Immunohistochemical analysis of xenograft tumor tissues. Scale bar: 100 μm. ns, p > 0.05; *p < 0.05; **p < 0.01; ***p < 0.001.

An s.c. xenograft mouse model was established to test the possibility that AB+ promotes survival of recipient cells *in vivo*. Mice were i.v. treated with saline (control), AB0, or AB+, then injected with 2×10^7^ SKM1 cells, then treated with Ara-C, and tumor volume was measured at 4-day intervals. Tumor growth rate was significantly higher in AB+ than in AB0 or control group (**Figure 4A**). Mice were sacrificed on day 28, and primary tumor was excised and weighed. Tumor volume and weight were significantly higher in AB+ than the other two groups (**Figures 4B, C**, **S6A**). Western blotting analysis revealed significantly increased IGF2BP3, p-PI3K, p-AKT and BCL2 expression in AB+-treated tumor tissues (**Figure 4D**). Immunohistochemical analysis likewise showed increased IGF2BP3, c-Myc, p-AKT, and p-ERK expression in the AB+-treated group (**Figures 4E**, **S6B, C**).

### Patient ABs Deliver IGF2BP3 Into Recipient Cells

**Figure 5:**
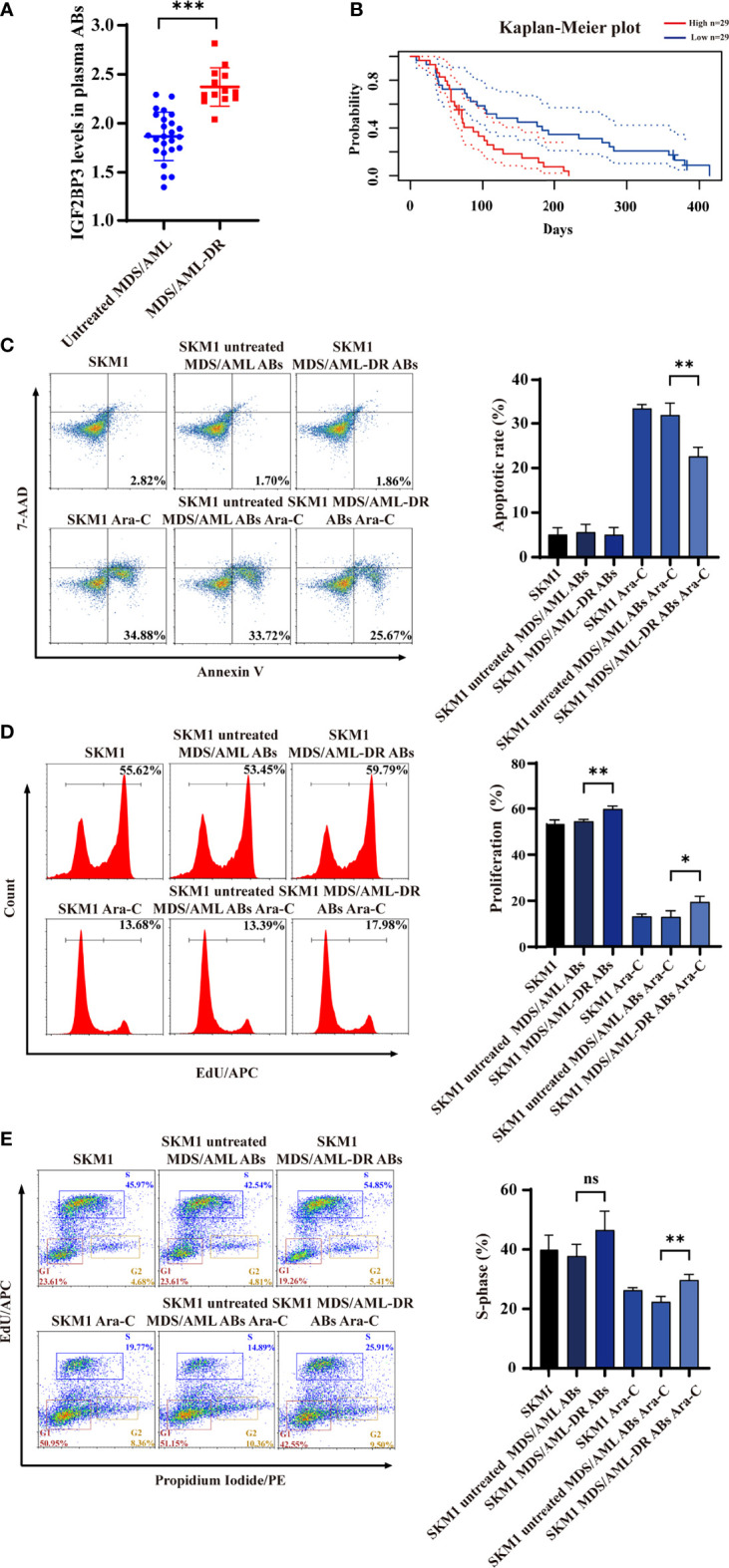
IGF2BP3 expression of ABs from clinical patient samples. **(A)** ELISA of IGF2BP3 level. **(B)** Prognosis of MDS/AML patients with various IGF2BP3 levels, analyzed using PrognoScan database. **(C-E)** SKM1 cells were treated with patient-derived ABs, and apoptosis **(C)**, proliferation **(D)** and cell cycle **(E)** were evaluated. ns, p > 0.05; *p < 0.05; **p < 0.01; ***p < 0.001.

ABs from plasma of untreated MDS/AML patients and chemotherapy-resistant patients (MDS/AML-DR) (**Table S1**) were obtained by gradient centrifugation. IGF2BP3 content was significantly higher in ABs from the latter vs. former group (**Figure 5A**). Analysis using PrognoScan database (25) indicated correlation between high IGF2BP3 expression and poor prognosis (**Figure 5B**). Incubation of SKM1 cells with ABs from MDS/AML-DR patients resulted in reduced apoptosis (**Figure 5C**), enhanced proliferation (**Figure 5D**), and promotion of cell cycle G1/S phase (**Figure 5E**).

## Discussion

Chemotherapy is the preferred treatment for MDS/AML patients. However, prolonged chemotherapy often leads to development of varying degrees of drug resistance, which has major effects on quality of life and recovery process (26). Studies during the past decade indicate that EVs can mediate drug resistance by several mechanisms. For example, they may remove drugs taken up by cells, thereby reducing effective concentration. Exosomes derived from drug-resistant breast cancer cells induced drug-resistant phenotype by transferring drug-resistance-related gene MDR-1 and P-glycoprotein (27). Tumor cell-derived EVs can be used to promote tumor immune escape by carrying membrane proteins that interfere with binding of immune cells. Metastatic melanoma promotes its own development by releasing a large number of EVs with PD-L1 on membrane surface to inhibit CD8+ T cell function (28). EVs from drug-resistant tumor cells can deliver mRNA, miRNA, long noncoding RNA, or proteins to induce resistance in sensitive recipient cells (29–31). Yang et al. (32) demonstrated that exosomes derived from gemcitabine-resistant pancreatic cancer stem cells mediated horizontal transfer of drug-resistant traits to sensitive pancreatic cancer cells by delivering miR-210 (32). Exosomes derived from gemcitabine-resistant human A549 lung cells were internalized by recipient cells, enabling transfer of miR-222-3p. Exosomal miR-222-3p enhanced proliferation, gemcitabine resistance, migration, invasion, and anti-anoikis of parental sensitive cells by directly targeting SOCS3 promoter (33). Despite the above reports, the role of ABs in mediating drug resistance remains poorly understood.

We identified and characterized ABs from two types of myeloid cells. Only ABs produced by Ara-C-treated cells enhanced proliferation and anti-apoptosis capacity of recipient cells. Drug treatment may result in differing “packages” of ABs, providing a basis for their various biological functions. ABs are phospholipid bilayer-enclosed EVs, similar to exosomes, and are likewise able to carry a variety of biomolecules, including DNA, RNA, proteins, and fatty acids (13). We used a proteomic approach to identify 1577 proteins that were differentially expressed in the two types of ABs. Chemotherapeutic agents were shown to significantly increase content of IGF2BP3, a potential oncoprotein, in ABs from Ara-C-treated cells.

IGF2BP3 has been characterized as an oncoprotein and a useful biomarker for a variety of cancers, including adenocarcinoma (34), intrahepatic cholangiocarcinoma (35), squamous esophageal cancer (36), and colon cancer (37). Our analysis of clinical samples revealed an inverse correlation of high IGF2BP3 level with MDS/AML patient survival time. IGF2BP3 binds specific mRNAs, including IGF2, in cytoplasm, and affects their stability and translation (38). In human K562 chronic myeloid leukemia (CML) cells, IGF2BP3 enhanced cell survival following ionizing radiation (IR) therapy by binding to IGF2 mRNA, whereas IGF2BP3 knockdown inhibited cell proliferation, suggesting potential use of IGF2BP3 as a novel drug target for promoting CML sensitivity to IR therapy (39). IGF2BP3 can bind to mRNAs other than IGF2 mRNA, and modulate their expression. In hepatocellular carcinoma and glioblastoma, upregulation of IGF2BP3 increased c-Myc expression and activated PI3K-AKT pathway, thereby affecting cell proliferation, cell cycle, and apoptosis (40, 41). Samanta et al. (42) reported that IGF2BP3 bound to mRNA of breast cancer resistance protein (BCRP; also known as ABCG2), a type of ATP-binding cassette (ABC) transporter and a major effector of drug resistance, thereby modulating its expression and promoting drug resistance of breast cancer cells (42). In ovarian cancer, combined high expression of IGF2BP3 and of Lin28B, another RNA-binding protein, was strongly associated with chemoresistance and poor disease outcome, based on elevated expression of hCTR1, a copper transporter involved in platinum uptake (43). Yoshino etal. (36) identified IGF2BP3 as a radioresistance factor in squamous esophageal cancer cells (36).

We observed that IGF2BP3 carried by ABs was phagocytosed by recipient cells. Following phagocytosis, IGF2BP3 promoted expression of oncoprotein c-Myc, which in turn activated PI3K-AKT and P42-44 MAPK signaling pathways. JQ-1, a bromine terminal inhibitor, was reported to inhibit IGF2BP3 function (24), and the inhibitory effect of JQ-1 on IGF2BP3 was confirmed in our study. Activation of these pathways further promoted cell proliferation, anti-apoptotic capacity, and drug resistance of recipient cells. ABs from non-drug-responsive MDS/AML patients reduced Ara-C sensitivity of recipient cells. Studies using our *in vivo* mouse model confirmed these findings.

Still, the molecular mechanism of drug resistance mediated by AB-encapsulated IGF2BP3 remains unclear. IGF2BP3 contains several K homology (KH) domains: evolutionarily conserved modules that play important roles in RNA binding and are involved in RNA synthesis and metabolism. IGF2BP3 is capable of binding to many mRNAs besides IGF2 mRNA. We propose two mechanisms whereby IGF2BP3 mediates drug resistance in recipient cells: (i) IGF2BP3, together with its binding mRNAs that encode specific proteins, are jointly packaged into ABs and endocytosed into recipient cells, where the mRNAs are translated and resulting proteins have key functions in drug resistance; (ii) IGF2BP3 itself is endocytosed *via* ABs, binds to specific endogenous mRNAs in recipient cells, promotes their translation, and thereby enhances drug resistance. These possible mechanisms are the subject of ongoing studies.

## Conclusion

ABs are important vehicles of intercellular communication. The protein IGF2BP3 was shown to be enriched in ABs secreted by Ara-C-resistant cells, phagocytosed by Ara-C-sensitive cells, and to help enhance the ability of these cells to evade apoptosis under chemotherapy. Cargoes (particularly IGF2BP3) that are overexpressed in donor cells and packaged into ABs are therefore potentially useful targets for more effective chemotherapy of MDS or AML patients.

## Data Availability Statement

The original contributions presented in the study are included in the article/**Supplementary Material**. Further inquiries can be directed to the corresponding authors.

## Ethics Statement

The studies involving human participants were reviewed and approved by Research Ethics Committee of Northwest University. The patients/participants provided their written informed consent to participate in this study. The animal study was reviewed and approved by Animal Care and Use Committee guidelines of Northwest University.

## Author Contributions

JG, XP, HL, and JB performed experiments. JG and HL analyzed data. XL and YW provided expertise. YW provided clinical samples. XL and YW designed and supervised the project. YW, JG, and XL wrote the manuscript. All authors contributed to the article and approved the submitted version.

## Funding

The study was supported by the National Science Foundation of China (Grants 81770123, 32071274, 82100148, 82000130), Science Foundation for Distinguished Young Scholars of Shaanxi Province (2021JC-39), Natural Science Foundation of Shaanxi Province (2019JZ-22, 2021SF-294), and Youth Innovation Team of Shaanxi Universities.

## Conflict of Interest

The authors declare that the research was conducted in the absence of any commercial or financial relationships that could be construed as a potential conflict of interest.

## Publisher’s Note

All claims expressed in this article are solely those of the authors and do not necessarily represent those of their affiliated organizations, or those of the publisher, the editors and the reviewers. Any product that may be evaluated in this article, or claim that may be made by its manufacturer, is not guaranteed or endorsed by the publisher.
