# Supplementary material for: Transfer of IGF2BP3 Through Ara-C-Induced Apoptotic Bodies Promotes Survival of Recipient Cells
Source: Front Oncol. 2022 May 9;12:801226. doi: 10.3389/fonc.2022.801226 (PMC9124970; doi:10.3389/fonc.2022.801226)
Supplement: Supplementary file 1 [file DataSheet_1.docx]

Supplementary Material

**Figure 1**


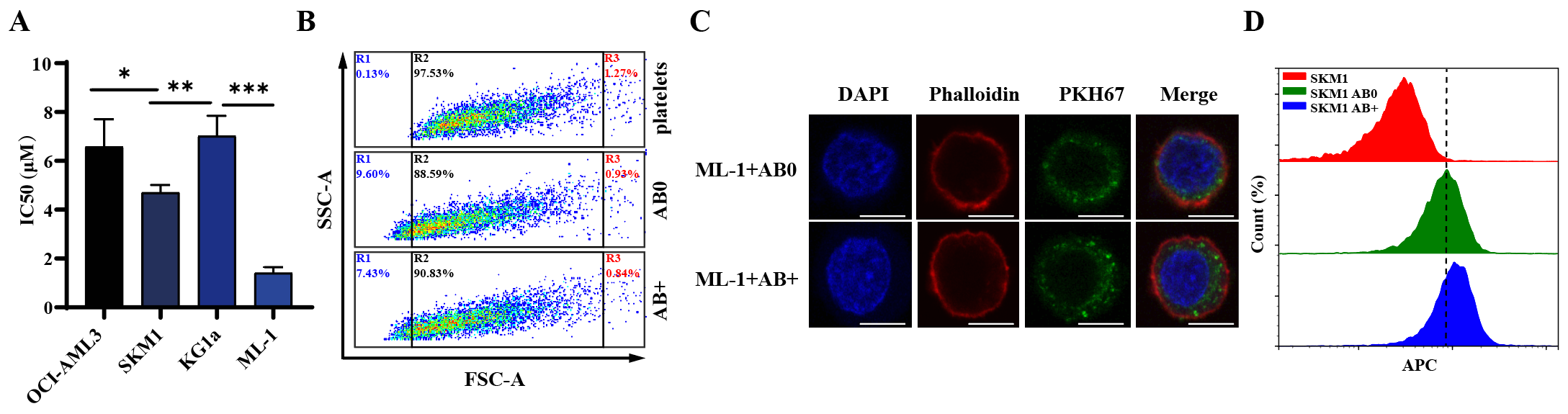


**Supplementary Fig 1. Characterization of apoptotic bodies.** (A) The IC50 of OCI-AML3, SKM1, KG1a and ML-1 cells to Ara-C was detected by CCK-8. (B) Flow cytometry was used to analyze the size of AB0 and AB+. Platelets were used as control to gate ABs (1-4 µm, gate R2). ABs derived from different groups of microparticles (gate R1), ABs (gate R2) and cells (gate R3) were shown. (C) Confocal microscopy image showed the internalization of ABs. ABs was dyed with PKH67. Scale bar: 10 µm. (D) AB0 or AB+ were stained with 50 μg Annexin V and then incubated with SKM1 cells for 30min, endocytosis efficiency was detected by flow cytometry.

**Figure2**


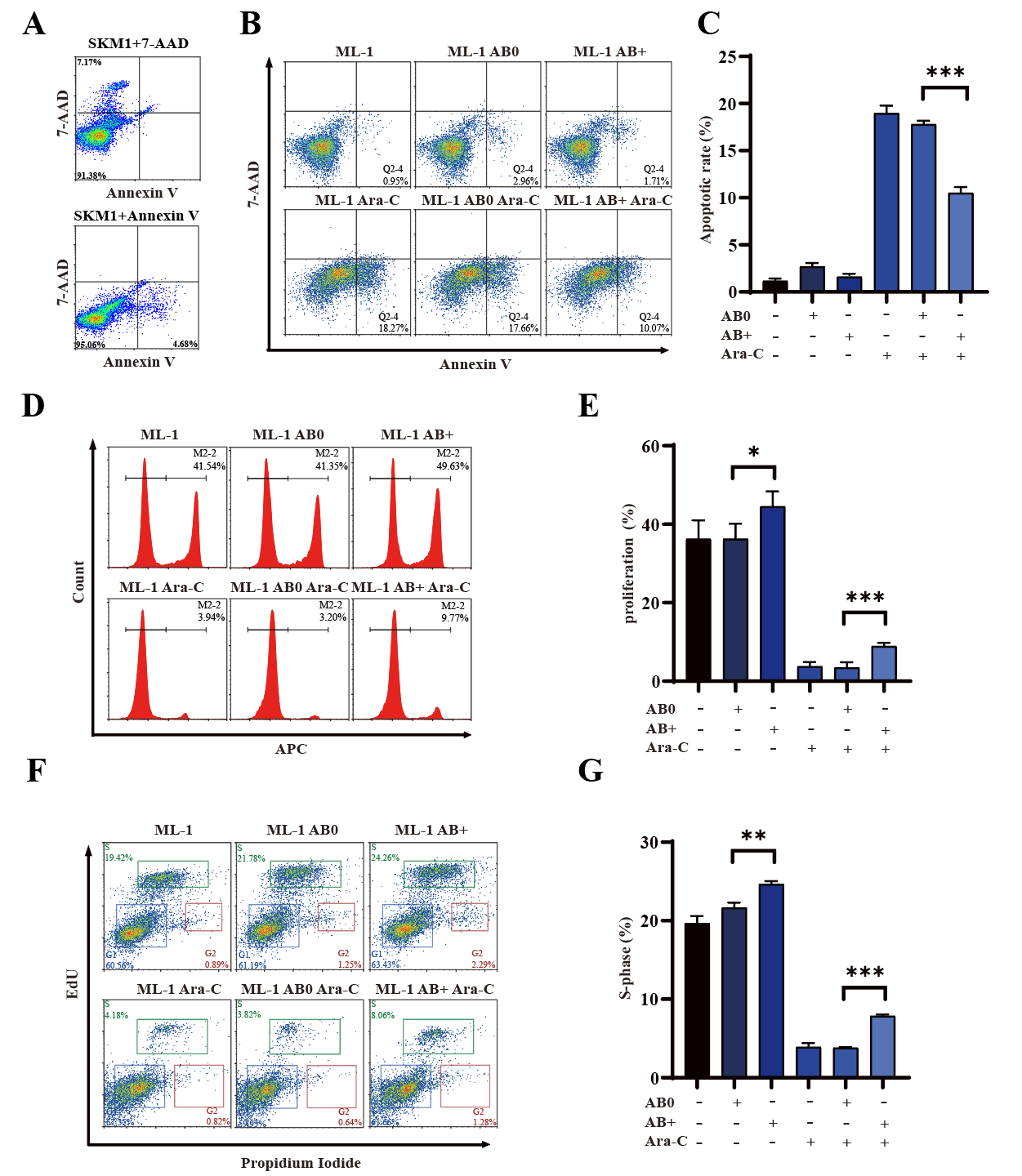


Supplementary Fig 2. (A) Gating strategy was shown. SKM1 cells were single-stained with 7-AAD or Annexin V. (B-G) ML-1 cells were pre-incubated with AB0 or AB+ for 8 h, then treated with 2 μM Ara-C for 48 h. Controls were untreated cells, and cells treated with AB0 or AB+ but not Ara-C. Apoptosis (B-C), proliferation (D-E), and cell cycle (F-G) were evaluated by flow cytometry.

**Figure3**


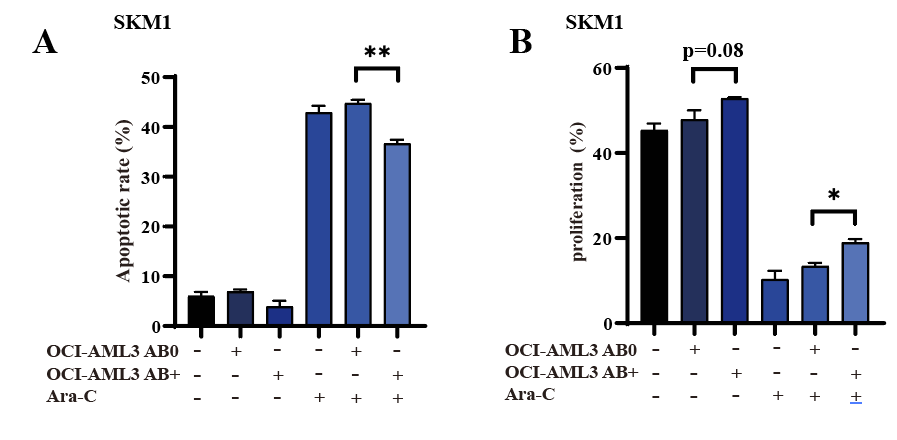


**Supplementary Fig3.** (A) OCI-AML3 cells were used as donor cells. SKM1 cells were pre-incubated with AB0 or Ara-C induced AB+ for 8h, and treated with Ara-C for 48h. Controls were untreated cells, and cells treated with AB0 or AB+ but not Ara-C. Apoptosis (A) and Cell proliferation (B) were evaluated by flow cytometry of SKM1 cells was detected by flow cytometry.

**Figure 4**


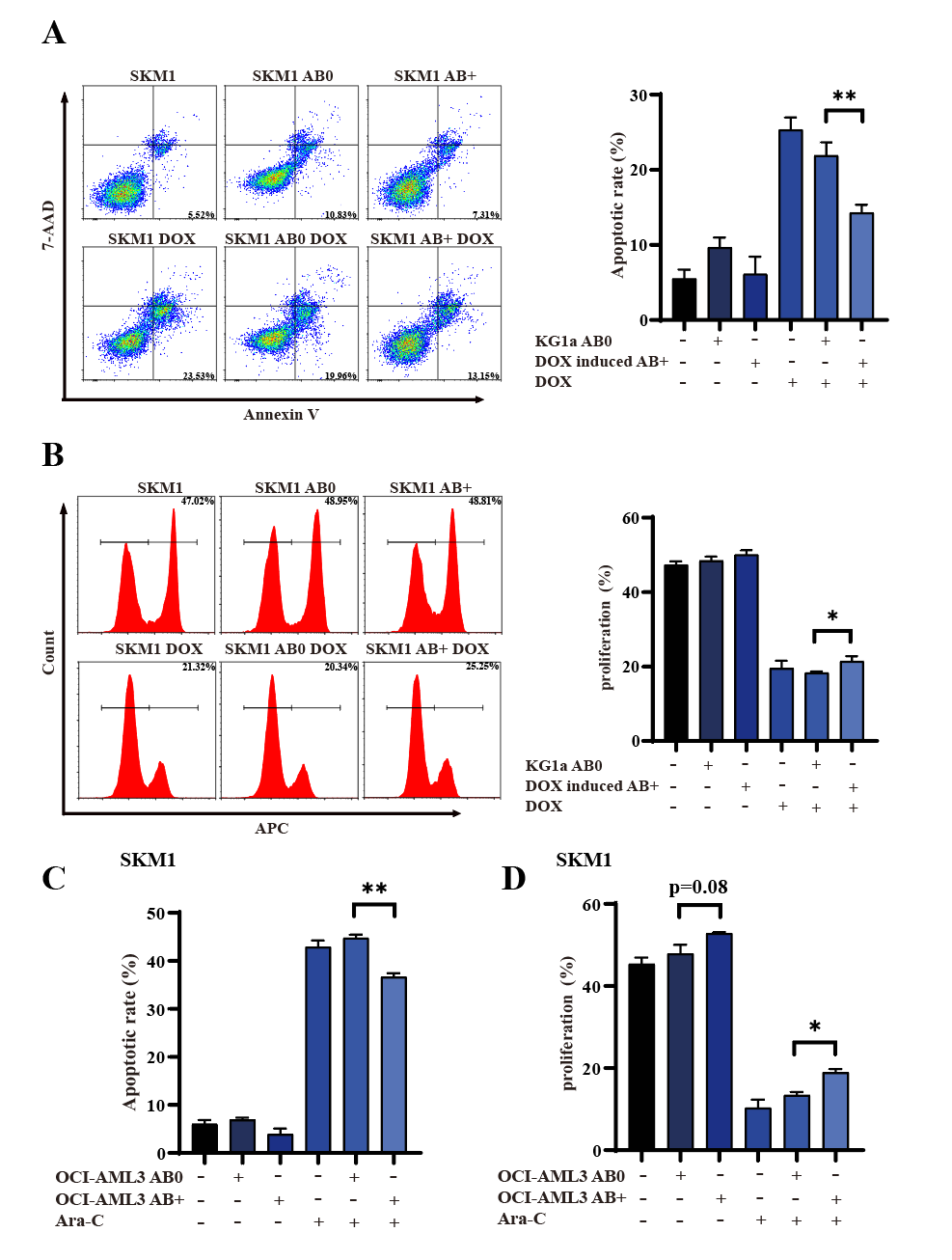


**Supplementary Fig 4.** (A) SKM1 cells were pre-incubated with AB0 or DOX induced AB+ for 8h, and treated with1 μM DOX for 48h. Controls were untreated cells, and cells treated with AB0 or AB+ but not DOX. Apoptosis (A) and Cell proliferation (B) were evaluated by flow cytometry.

**Figure 5**


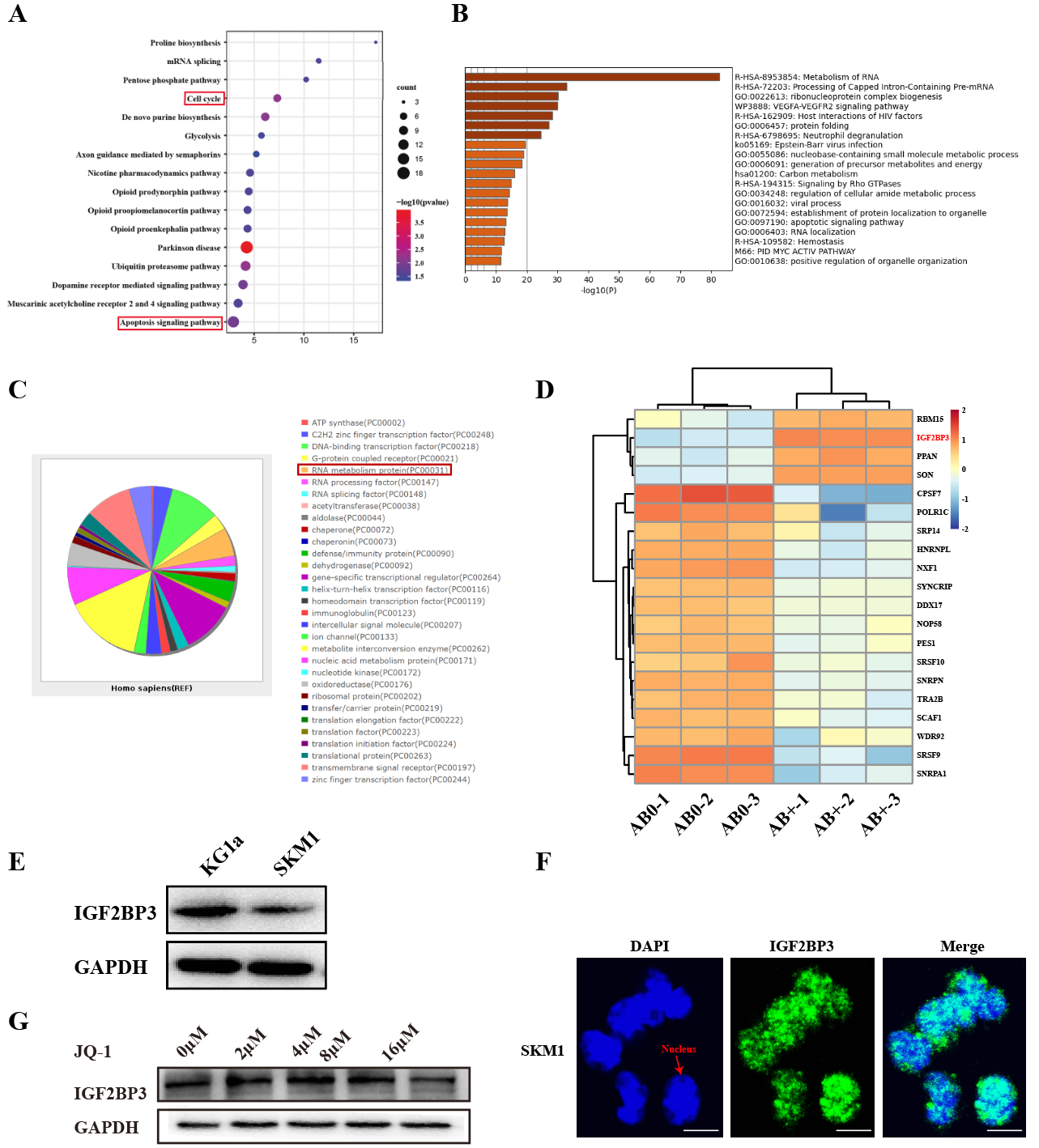


**Supplementary Fig 5.** (A) KEGG analysis of possible role of differential proteins in the signaling pathway. (B) The biological functions of differential proteins in AB0 and AB+ were analyzed by Metascape database. (C) Differentially expressed proteins were classified by the Gene Ontology Resource, RNA metabolism-related proteins were enriched. (D) The contents of top 20 RNA metabolism-related proteins in AB0 and AB+. (E) The expression of IGF2BP3 in KG1a and SKM1 cells were detected by western blotting. (F) Confocal microscopy image showing the location of IGF2BP3. Scale bar: 10 µm. (G) Different concentration of JQ-1, IGF2BP3 inhibitor, was added to SKM1 and the expression of IGF2BP3 was detected by western blotting.

**Figure6**


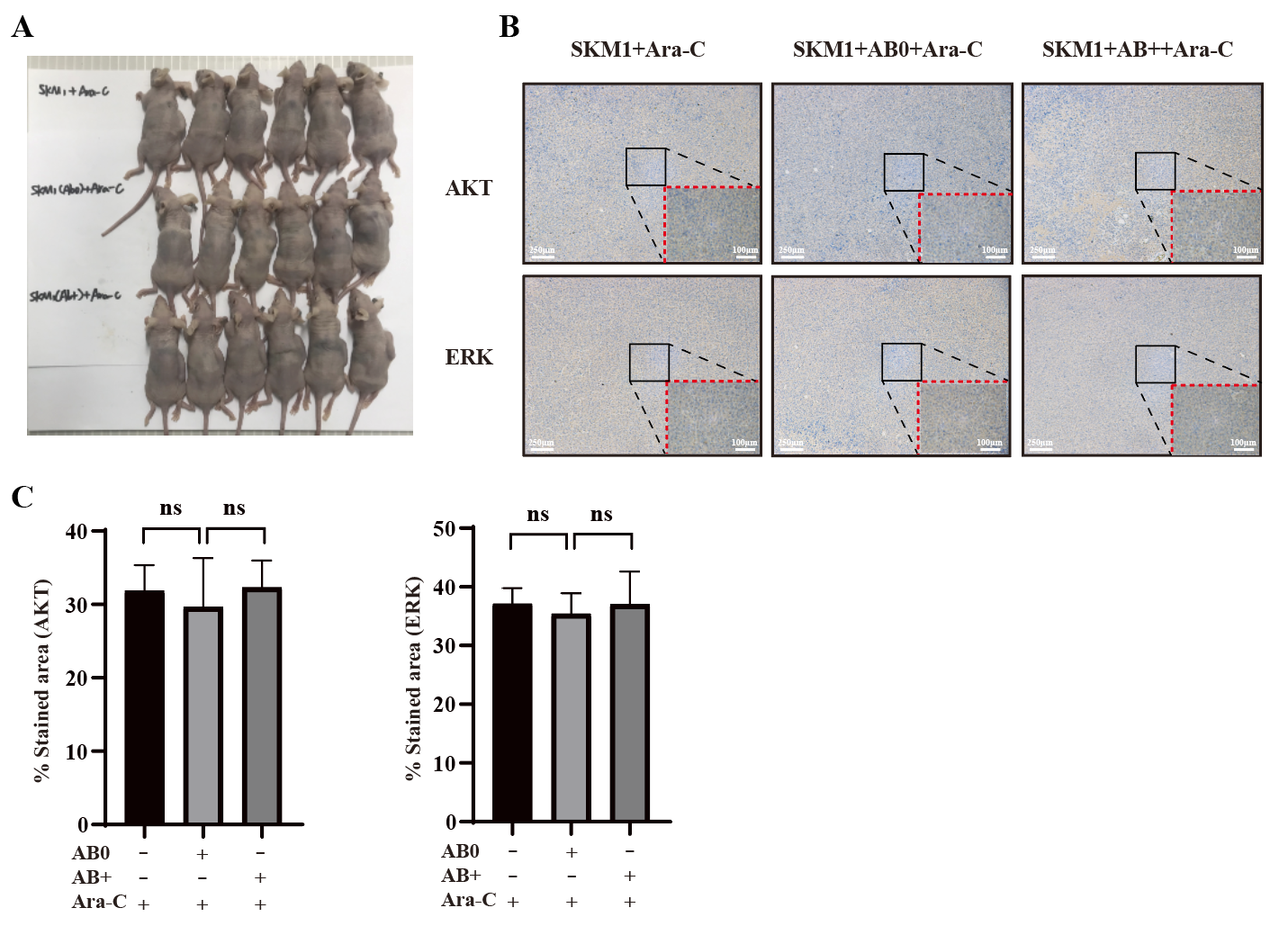


**Supplementary Fig 6.** (A) Six-to-8-wk-old mice were injected s.c. with 2×10^7^ SKM1 cells, and following tumor formation three experimental groups were i.v. injected respectively with PBS, 50 μg AB0, and 50 μg AB+. (B) Immunohistochemical analysis was performed to detect the expression of AKT and ERK in different groups. Scale, 100 μm. (C) Quantification of immunohistochemical results.

**Supplementary Tab 1.** Clinical sample patients information.

| Diagnosis | Age (yr) | Gender | Cytogenetics | BM cellularity | Marrow blast count | Ara-C response | WBC  (10^9^/L) |
| --- | --- | --- | --- | --- | --- | --- | --- |
| AML | 78 | M | t(8, 21) | normal | ＞20% | R | 3.41 |
| AML | 64 | M | normal | normal | 46% | R | 68.34 |
| MDS | 76 | M | normal | hyper | 73.5% | R | 1.39 |
| AML | 58 | M | t(8, 21) | normal | 29% | R | 43.23 |
| MDS | 59 | M | normal | hyper | 56% | R | 2.1 |
| AML | 70 | M | normal | normal | 44% | R | 25.33 |
| MDS | 30 | M | normal | normal | 25% | R | 7.05 |
| MDS | 46 | F | normal | normal | ＞20% | R | 1.28 |
| MDS | 57 | M | +8 | hyper | 12.4% | R | 1.82 |
| MDS | 39 | M | normal | hyper | 33.2% | R | 2.24 |
| MDS | 64 | M | normal | normal | 48.5% | R | 2.77 |
| MDS | 71 | M | normal | normal | 10.5% | R | 1.98 |
| AML | 72 | F | normal | normal | 33% | R | 28.87 |
| MDS | 66 | M | normal | normal | 2.5% | R | 11.11 |
| MDS | 42 | M | t(8, 21), +8 | hyper | 34.5% | R | 3.47 |
| MDS | 71 | M | normal | normal | 31% | R | 2.24 |
| AML | 69 | M | normal | hyper | ＜30% | R | 91 |
| MDS | 77 | F | normal | normal | 8.5% | R | 2.46 |
| AML | 46 | M | normal | normal | 33.8% | R | 15.78 |
| AML | 61 | F | normal | hyper | 27.89% | R | 21.18 |
| AML | 53 | M | t(8, 21) | hyper | 21.86% | R | 13.38 |
| AML | 66 | M | normal | normal | 56.12% | R | 21.83 |
| MDS | 32 | M | normal | normal | 41.25% | R | 12.37 |
| MDS | 58 | F | normal | normal | 56.5% | R | 2.35 |
| AML | 49 | M | normal | normal | 45.1% | R | 12.27 |
| AML | 68 | M | normal | hyper | 6% | NR | 25.93 |
| AML | 53 | F | normal | normal | 73.5% | NR | 8.4 |
| AML | 38 | F | normal | normal | 32.5% | NR | 20.72 |
| AML | 55 | M | normal | hyper | 62% | NR | 14.27 |
| AML | 72 | M | normal | normal | 49% | NR | 3.79 |
| AML | 15 | M | normal | normal | 34% | NR | 28.1 |
| AML | 45 | F | t(8, 21) | hyper | 23% | NR | 11.16 |
| AML | 56 | F | normal | hyper | 11% | NR | 3.77 |
| AML | 48 | F | normal | hyper | 24.5% | NR | 1.3 |
| AML | 34 | M | normal | normal | 26% | NR | 99.43 |
| AML | 67 | M | normal | hyper | 75% | NR | 234.56 |
| AML | 49 | M | normal | hyper | 32.5% | NR | 28.87 |
